# Association of vasopressors with mortality in critically ill patients with COVID-19: a systematic review and meta-analysis

**DOI:** 10.1007/s44254-023-00013-7

**Published:** 2023-04-23

**Authors:** Maria Mermiri, Georgios Mavrovounis, Eleni Laou, Nikolaos Papagiannakis, Ioannis Pantazopoulos, Athanasios Chalkias

**Affiliations:** 1 Department of Anesthesiology, Faculty of Medicine, University of Thessaly, 41110 Larisa, Biopolis Greece; 2 Department of Emergency Medicine, Faculty of Medicine, University of Thessaly, Larisa, Greece; 3 Department of Anesthesiology, Agia Sophia Children’s Hospital, Athens, Greece; 4 First Department of Neurology, Medical School, Eginition University Hospital, National and Kapodistrian University of Athens, Athens, Greece; 5 Outcomes Research Consortium, Cleveland, OH 44195 USA

**Keywords:** Covid-19, Critically ill, Hemodynamics, Vasopressor, Intensive care, Mortality

## Abstract

Exogenous catecholamines may have pronounced side effects and affect physiological cascades. The aim of this study was to investigate the effect of vasopressors on mortality of critically ill patients with coronavirus disease 2019 (COVID-19). A systematic search of PubMed, Scopus, and ClinicalTrials.gov was conducted for relevant articles until December 2022. Eligibility criteria were randomized controlled and non-randomized trials. The primary outcome was in-hospital and 30-day mortality. The quality of studies was assessed using the Methodological Index for Non-Randomized Studies (MINORS) tool, while paired meta-analysis was used to estimate the pooled risk ratios (RR) along with their 95% Confidence Interval (95% CI). Analyses of 22 studies (*n* = 8034) revealed that vasopressor use is associated with mortality compared to no vasopressor therapy [RR (95%CI): 4.30 (3.21, 5.75); *p* < 0.001]. In-hospital and 30-day mortality are significantly higher in patients who receive vasopressors [RR (95%CI): 4.60 (2.47, 8.55); *p* < 0.001 and RR (95%CI): 2.97 (1.72, 5.14); *p* < 0.001, respectively]. Also, analyses of data from 10 studies (*n* = 3519) revealed that vasopressor use is associated with acute kidney injury [RR (95%CI): 3.17 (2.21, 4.54); *p* < 0.001]. In conclusion, current use of vasopressors in critically ill patients with COVID-19 may be associated with higher in-hospital mortality, 30-day mortality, and incidence rate of acute kidney injury. Further research is required to estimate the correlation of specific vasopressor characteristics (type, timing, dose, combination) with adverse effects and mortality in this population.

**Graphical Abstract:**

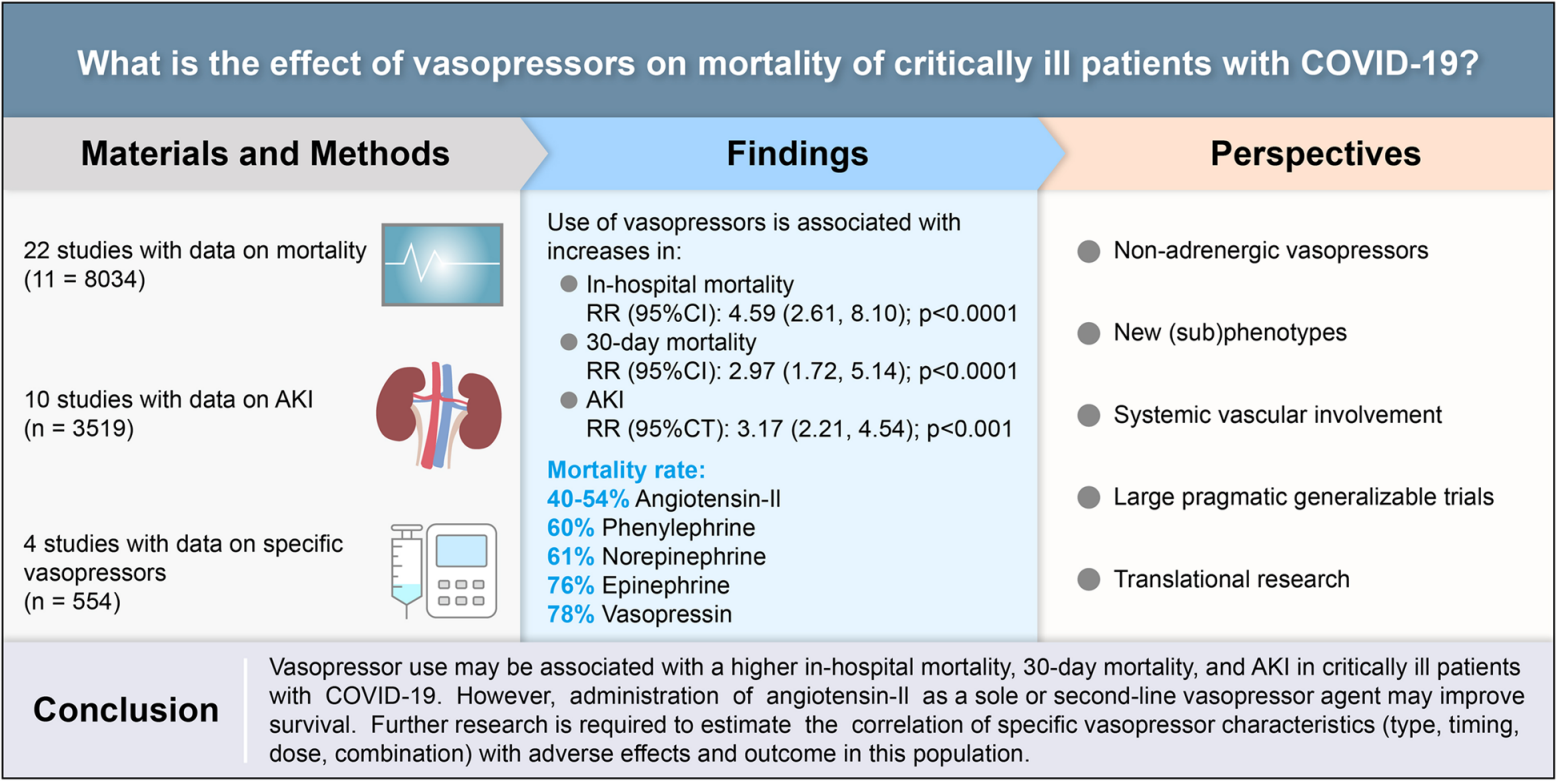

**Supplementary Information:**

The online version contains supplementary material available at 10.1007/s44254-023-00013-7.

## Introduction

Mounting evidence suggest that coronavirus disease 2019 (COVID-19) should be perceived as a new entity with its own characteristics and distinct pathophysiology, including complex immuno-inflammatory, thrombotic, and parenchymal derangements [1]. The cytokine storm and the dysregulation of host response are more severe in COVID-19-related acute respiratory distress syndrome (ARDS) than in ARDS of other causes [2–4]. SARS-CoV-2 not only infects the respiratory tract, but also injures the vascular endothelium and epithelium [5, 6].

Most critically ill patients with COVID-19 need hemodynamic support that may still be guided by the current, non-covid, surviving sepsis campaign guidelines recommending the use of vasopressors to optimize mean arterial pressure (MAP) and cardiac output and provide adequate organ perfusion [7–9]. Most of these medications improve the hemodynamic function through enhancement of the adrenergic pathway; however, they may have important side-effects due to excessive adrenergic stimulation [10–12]. Of note, exogenous catecholamines can have a pronounced impact on inflammation and immunosuppression, metabolism, endothelial lesion, platelet activation, and coagulation [13]. As critically ill patients with COVID-19 are characterized by a similar pathophysiology, exogenous vasopressors could further dysregulate their physiological cascades and aggravate outcome [14].

We therefore performed a systematic review and meta-analysis to investigate the effect of vasopressors on mortality of critically ill patients with COVID-19.

## Material and methods

The protocol was registered in the PROSPERO international prospective register of systematic reviews on 13 December 2021 (CRD42021297595). This systematic review and meta-analysis was designed according to the preferred reporting items for systematic reviews and meta-analyses (PRISMA) checklist (Additional file 1: Appendix A) [15].

### Inclusion and exclusion criteria

The inclusion criteria of the current systematic review and meta-analysis were: (1) randomized controlled trials (RCTs) and observational studies; (2) critically ill patients admitted to the intensive care (ICU) or high dependency unit (HDU), including patients admitted through the Emergency Department (ED); (3) adults (≥ 18 years old) hospitalized primarily for COVID-19; (4) SARS-CoV-2 infection confirmed by reverse transcription polymerase chain reaction test of nasopharyngeal or oropharyngeal samples; and (5) vasopressor *vs.* no vasopressor administration. We excluded animal studies, case reports, review papers, editorials, abstracts, white papers, and non-English literature. We also excluded studies about pediatric patients and non-ICU/HDU/ED patients.

### Outcomes of interest and data extraction

The primary outcome was in-hospital and 30-day mortality. Secondary outcome was to investigate (1) the hemodynamic profiles of patients at first measuring point and after six hours [heart rate, MAP, central venous pressure (CVP), urinary output, blood lactate levels, cardiac output or cardiac index, systemic vascular resistance index, central venous oxygen saturation, oxygen delivery index, and oxygen consumption index]; (2) the number of participants who achieved the target MAP; (3) time to achieve the target MAP; (4) adverse events including arrhythmia, acute myocardial infarction, cardiac arrest, acute mesenteric ischemia, digital ischemia, acute kidney injury (AKI); (5) vasopressor-free days; (6) ICU or HDU length of stay; (7) duration of mechanical ventilation; (8) ventilator free days; (9) hospital length of stay; and (10) all-cause mortality at 90-days.

The data from each study were extracted by two independent authors (MM, GM) with a customized format. Any disagreements between the two independent authors were resolved by four other authors (EL, IP, NP, AC). Publication details (authors, year), study information (design, population, department of admission, follow-up, inclusion–exclusion criteria, number of cases/cohort-size, and subgroups), hemodynamic profile (heart rate, MAP, CVP, urinary output, blood lactate levels, cardiac output or cardiac index, systemic vascular resistance index, central venous oxygen saturation, oxygen delivery index, oxygen consumption index) at first measuring point and six hours after vasopressor use, the number of participants who achieved the target MAP and time to achieve the target MAP, adverse events, vasopressor-free days, ICU length of stay, hospital length of stay, duration of mechanical ventilation, ventilator-free days, all-cause mortality in all groups at 28 or 30 days, and all-cause mortality at 90 days were extracted in a pre-designed excel spreadsheet. The definition used for AKI and the mortality follow-up timepoints for each study are presented in Additional file 3: Appendix C1. Authors of studies with missing data were contacted in an attempt to obtain relevant data.

### Search strategy

The search strategy was intended to explore all available published and unpublished studies from January 2020 to January 2022. A comprehensive initial search was employed in PubMed (MEDLINE), Scopus, and ClinicalTrials.gov databases by two independent investigators (MM, GM) followed by an analysis of the text words contained in Title/Abstract and indexed terms. A second search was conducted by combining free text words (vasopressor, epinephrine, norepinephrine, phenylephrine, vasopressin, dopamine, angiotensin-II, covid-19, critically ill, intensive care) and indexed terms with Boolean operators. Finally, a third search was conducted with the reference lists of all identified reports and articles for additional studies. After the initial data was compiled, a refresh repeat search until December 31, 2022 was performed. Additional file 2 (Appendix B) presents the exact search algorithm used for all databases.

### Assessment of methodological quality

Articles identified for retrieval were assessed by two independent authors (MM, GM) for methodological quality before inclusion in the review using standardized critical appraisal tools. The quality of the included observational studies was assessed using the Methodological Index for Non-Randomized Studies (MINORS) tool [16], while the Risk of Bias 2.0 (RoB 2.0) tool was used for RCTs [17]. Any disagreements between the authors appraising the articles were resolved through discussion with the other authors.

### Data analysis and synthesis

A paired meta-analysis was used to estimate the pooled risk ratios (RR) along with their 95% Confidence Interval (95% CI). Based on the presence of statistical heterogeneity, the meta-analysis was conducted according to fixed- or random effect models. The statistical heterogeneity was estimated by the use of the Cochran’s Q and *I*^2^ indices. When *I*^2^ > 50% and/or P_Q_ < 0.10, the random effects model was used, otherwise the fixed effects model was implemented [18]. Funnel plots as well as the Begg’s test were used to determine the existence of publication bias [19, 20]. The statistical significance was set at *p* < 0.05. All statistical analyses were performed in Review Manager (Rev-Man) [Computer program], Version 5.3. Copenhagen: The Nordic Cochrane Centre, The Cochrane Collaboration, 2014.

## Results

**Fig. 1:**
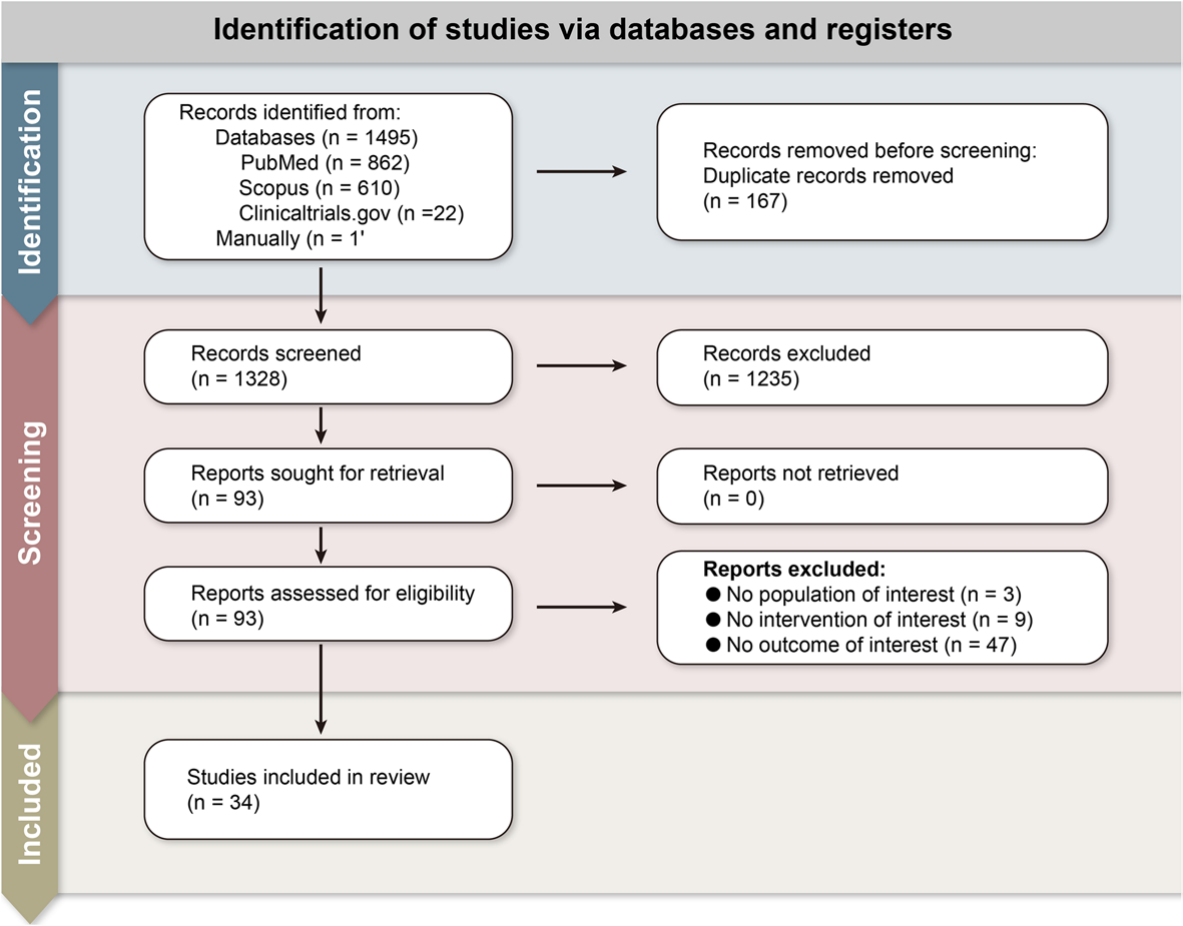
Preferred Reporting Items for Systematic Reviews and Meta-Analyses (PRISMA) diagram

Altogether, 1495 relevant citations were identified and screened, while 93 studies were included in our final assessment for possible data extraction (Fig. 1). In total, data extraction was possible in 34 studies [21–54].

### Study characteristics

All the 34 included studies were observational in their design [21–54]. Twenty studies included only patients admitted to the ICU [21, 23, 25, 26, 28, 31–33, 36–40, 44–48, 53, 54], five studies included patients admitted to a COVID-19-dedicated HDU [29, 30, 34, 35, 43], eight studies included patients who were admitted to both HDU and ICU [22, 24, 27, 41, 42, 49, 50, 52], and one study included Emergency Department patients who were later admitted either to ICU [51]. Thirty-one studies included data about patients who received *vs.* patients who did not receive vasopressors [21–38, 40–49, 51, 53, 54] and were included in review. Moreover, three studies included patients who received angiotensin-II [39, 50, 52] and, out of those, two compared the use of angiotensin-II with other vasopressors [50, 52] (Additional file 3: Appendix C2). Table 1 summarizes the main characteristics of the included studies.

**Table 1:** Main characteristics of the included studies

Authors Country, YOP Study Design	Department	Number of patients		Αge Mean ± SD / Median (IQR)	Μale (%) / Female (%)
		**Received vasopressors**	**No vasopressors**		
Ionescu et al. USA, 2021 Retrospective [21]	ICU	191	90	61 ± 13.9	154 (54.8%) / 127 (45.2%)
Pelayo et al. USA, 2020 Retrospective [22]	ICU + HDU	42	181	65.91 ± 14.95	116 (52%) / 107 (48%)
Lowe et al. UK, 2021 Retrospective [23]	ICU	49	32	57 ± 18	50 (61.7%) / 31 (38.3%)
Hansrivijit et al. USA, 2021 Retrospective [24]	ICU + HDU	53	230	64.1 ± 15.9	159 (56.2%) / 124 (43.8%)
Mesquida et al. Spain/Mexico/Brazil, 2021 Prospective [25]	ICU + IRCU	12	59	59 ± 13	51 (69.9%) / 22 (30.1%)
Ghosn et al. UAE, 2021 Retrospective [26]	ICU	66	44	50 (40–59)	98 (89.1%) / 12 (10.9%)
Farooqui et al. Saudi Arabia, 2021 Retrospective [27]	ICU + HDU	249	776	55.8 ± 18.52	582 (56.8%) / 443 (43.2%)
Neves et al. Brazil, 2021 Retrospective [28]	ICU	54	41	64.9 ± 15.1	61 (64.2%) / 34 (35.8%)
Bernardo et al. Portugal, 2021 Retrospective [29]	HDU	18	526	68.9 ± 17.9	298 (54.8%) / 246 (45.2%)
Hardenberg et al. Germany, 2021 Retrospective [30]	HDU	95	128	62 (51–75)	147 (65.9%) / 76 (34.1%)
Geri et al. France, 2021 Retrospective [31]	ICU	165	214	62 (53.69)	291 (76.8%) / 88 (23.2%)
Namendys-Silva et al. Mexico, 2021 Retrospective [32]	ICU	139	25	57.3 ± 13.7	114 (69.5%) / 50 (30.5%)
Auld et al. USA, 2021 Retrospective [33]	ICU	143	74	64 (54–73)	119 (54.8%) / 98 (45.2%)
Nabors et al. USA, 2021 Retrospective [34]	HDU	23	64	86 (80–105)	48 (55.2%) / 39 (44.8%)
Salacup et al. USA, 2021 Retrospective [35]	HDU	49	193	66 (58–76)	123 (50.8%) / 119 (49.2%)
Nasrulah et al. USA, 2021 Retrospective [36]	ICU	24	34	62 (54–73)	37 (63.8%) / 21 (36.2%)
Sjostorm et al. Sweden, 2021 Prospective [37]	ICU	40	13	59 (33–76)	39 (73.6%) / 14 (26.4%)
Ismail et al. UAE, 2021 Retrospective [38]	ICU	176	195	53 ± 13	314 (84.6%) / 57 (15.4%)
Osofu-Barko et al.^a^ USA, 2021 Retrospective [39]	ICU	10	Not available	64.5 ± 6.15	9 (90%) / 1 (10%)
Ramkumar et al. India, 2021 Prospective [40]	ICU	29	31	50 (37.5–63)	42 (70%) / 18 (30%)
Biccard et al. (ACCCOS) Africa^b^, 2021 Prospective [41]	ICU + HDU	931	2155	56 ± 16.11	1890 (60.6%) / 1228 (39.4%)
Mammen et al. India, 2021 Secondary analysis of RCT [42]	ICU + HDU	18	433	51 ± 12.4	346 (76.7%) / 105 (23.3%)
Andrade et al. USA, 2021 Retrospective [43]	HDU	63	221	67 ± 14.5	155 (54.6%) / 129 (45.4%)
Chand et al. USA, 2020 Retrospective [44]	ICU	233	67	58.2 ± 12.6	182 (60.7%) / 118 (39.3%)
Bezzera et al. Brazil, 2021 Retrospective [45]	ICU	325	99	66.42 ± 13.79	251 (59.2%) / 173 (40.8%)
Dang et al. USA, 2021 Retrospective [46]	ICU	63	26	65 (57–70)	52 (58.4%) / 37 (41.6%)
Gundogan et al. Turkey, 2021 Retrospective [47]	ICU	173	248	67 (57–76)	251 (59.6%) / 170 (40.4%)
Estella et al. Spain, 2021 Prospective [48]	ICU	419	3	63 (54–71)	305 (72.3%) / 117 (27.7%)
Gadhiya et al. USA, 2021 Retrospective [49]	ICU + HDU	53	230	64.1 ± 15.9	159 (56.2%) / 124 (43.8%)
Serpa Neto et al.a Multicentric study, 2022 Prospective [50]	ICU + HDU	65	67	61 (53–67)	105 (79.5%) / 27 (20.5%)
Brandão Neto et al. Brazil, 2021 Prospective [51]	ED and then transferred to ICU + HDU	179	327	60.1 ± 15.1	290 (57.3%) / 216 (42.7%)
Leisman et al.a USA, 2020 Retrospective [52]	ICU + HDU	10	19	56 ± 14	19 (65.5%) / 10 (34.5%)
Burrell et al. Australia, 2021 Prospective [53]	ICU	111	93	63.5 (53–72)	140 (68.6%) / 64 (31.4%)
Ramos et al. Multicentric study, 2022 Retrospective [54]	ICU	337	308	61.4 ± 16.6	387 (60%) / 258 (40%)

*YOP* Year of Publication, *SD* Standard Deviation, *IQR* Interquartile Range, *USA* United States of America, *ICU* Intensive Care Unit, *HDU* High Dependency Unit, *UK* United Kingdom, *IR3CU* Intensive Respiratory Care Unit, *UAE* United Arab Emirates, *ED* Emergency Department

^a^These studies included patients who received angiotensin-II

^b^10 African Countries

### Synthesis including all patients

#### Primary outcome

**Fig. 2:**
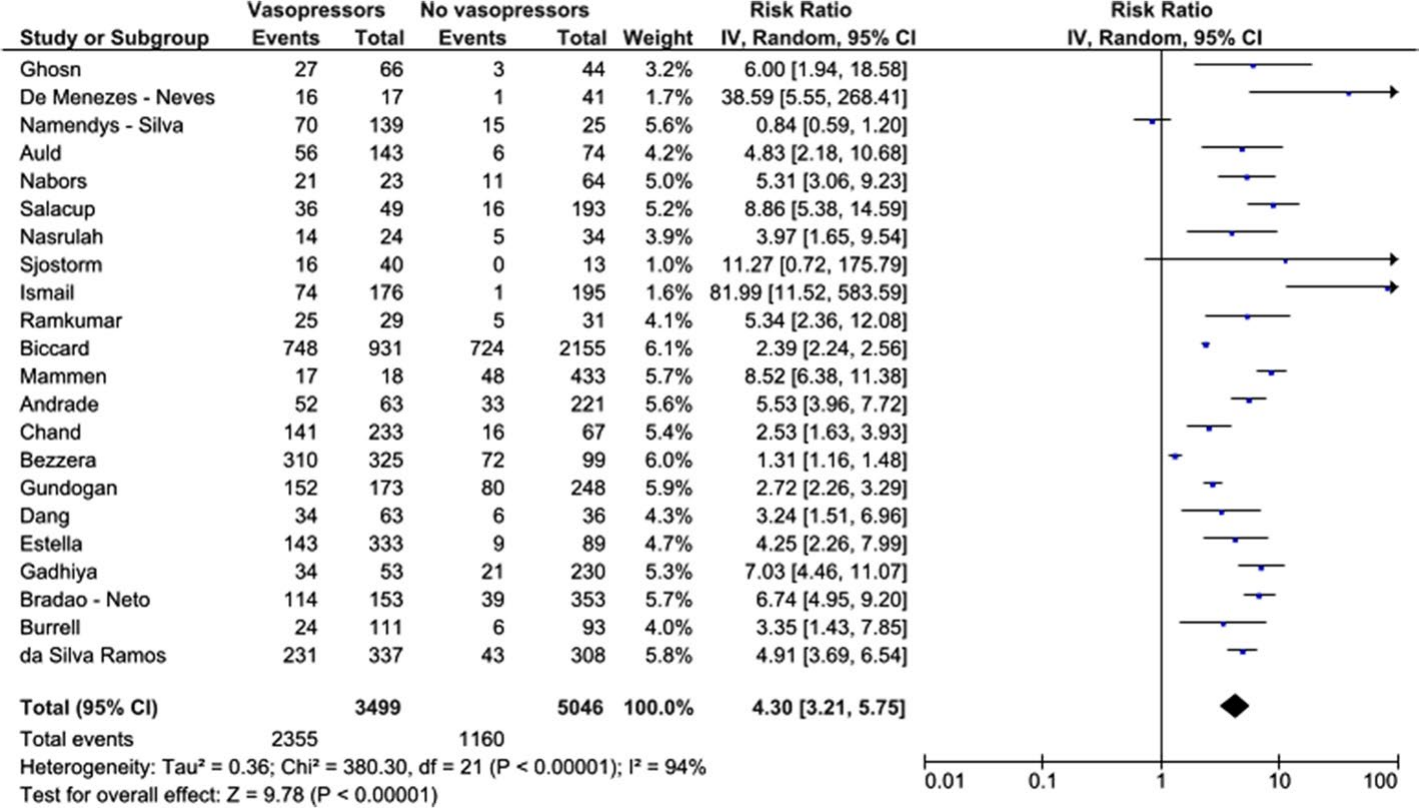
Effect of vasopressor use on mortality of critically ill patients with COVID-19

Twenty-two out of the 34 included studies provided data on in-hospital mortality in patients who received *vs.* patients who did not receive vasopressors, resulting in a total population of 8034 individuals [26, 28, 32–38, 40–49, 51, 53, 54]. Due to high heterogeneity (I^2^: 94%, P_Q_ < 0.001), the random-effects model was implemented. Vasopressor use is associated with mortality in patients who received vasopressors compared to those who did not receive vasopressor therapy [RR (95%CI): 4.30 (3.21, 5.75); *p* < 0.0001] (Fig. 2). Visual inspection of the funnel plot (Additional file 4: Appendix D1) and Begg’s test (*p* = 0.93) did not reveal significant publication bias.

We performed subgroup analyses based on the department of admission. The results for all three subgroups, namely ICU [RR (95%CI): 3.64 (2.44, 5.44); *p* < 0.0001], HDU [RR (95%CI): 6.25 (4.63, 8.44); *p* < 0.0001], and ICU + HDU [RR (95%CI): 5.52 (2.51, 12.15); *p* < 0.0001], remained statistically significant for higher mortality rates in patients who received vasopressors.

Subgroup analyses were also performed based on the mortality follow-up timepoints. Only the in-hospital and 30-day mortality subgroups had three or more studies that allowed data extraction and analysis. The in-hospital and 30-day mortality were significantly higher in patients who received vasopressors [RR (95%CI): 4.59 (2.61, 8.10); *p* < 0.0001 and RR (95%CI): 2.97 (1.72, 5.14); *p* < 0.0001, respectively].

#### Effect of major vasopressors on mortality

Four studies provided data on mortality based on the specific vasopressor(s) administered [39, 44, 50, 52]. The highest mortality rate was observed in patients treated with vasopressin or epinephrine (78% and 76%, respectively) [44]. Three of those studies investigated the role of angiotensin-II as a sole or second-, third-, fourth-, or fifth-line vasopressor agent [39, 50, 52]. These studies showed the lowest mortality rate. The relevant data are depicted in Table 2.

**Table 2:** Data on mortality based on major vasopressors

Authors Country, YOP Study Design	Intervention Group: Deaths / All (%)	Comparator Group: Deaths / All (%)
Chand et al. USA, 2020 Retrospective [44]	Any vasopressor support: 141 / 233 (61%) Norepinephrine ± other vasopressors: 138 / 226 (61%) Phenylephrine ± other vasopressors: 53 / 89 (60%) Vasopressin ± other vasopressors: 81 / 104 (78%) Epinephrine ± other vasopressors: 19 / 25 (76%)	No vasopressor support: 16 / 67 (24%)
Leisman et al. USA, 2020 Retrospective [52]	Angiotensin-II ± other vasopressors: 4 / 10 (40%)	Other vasopressors: 10 / 19 (53%)
Serpa Neto et al. Multicentric study, 2022 Prospective [50]	Angiotensin-II ± norepinephrine: 35 / 65 (54%)	Other vasopressors: 27 / 67 (40%)
Ofosu-Barko et al. USA, 2021 Retrospective [39]	Angiotensin-II + other vasopressors: 4 / 10 (40%)	-

#### Secondary outcomes – acute kidney injury

**Fig. 3:**
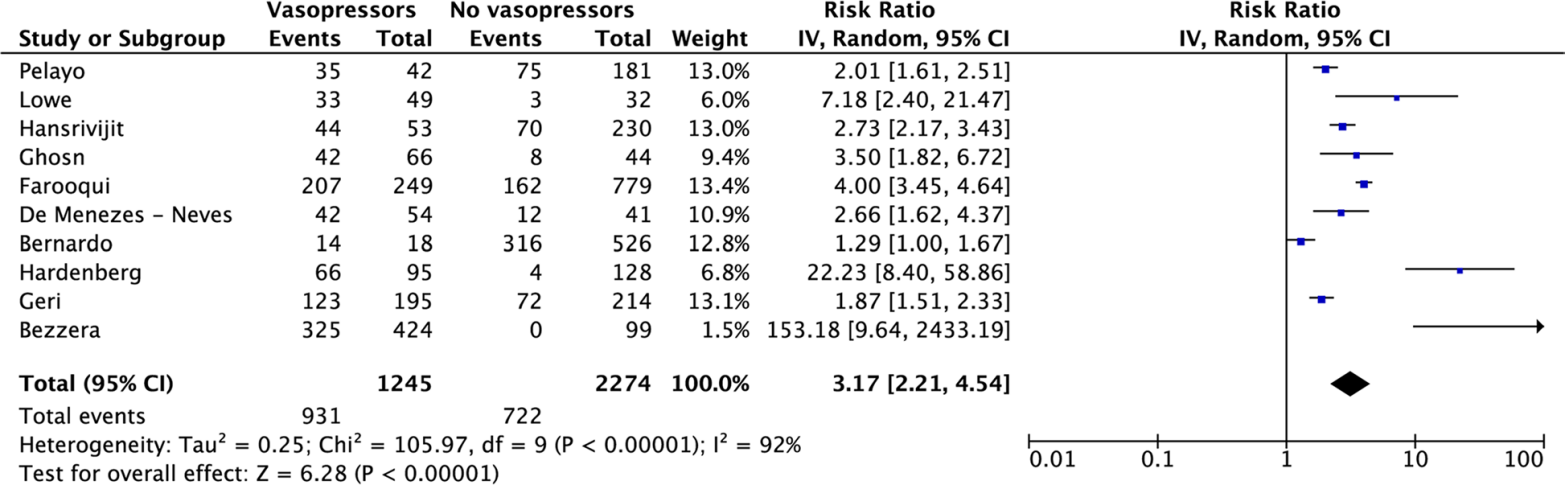
Effect of vasopressor use on the incidence of acute kidney injury

Ten studies provided data on AKI in patients who received *vs.* patients who did not receive vasopressors, resulting in a total population of 3519 individuals [22–24, 26–31, 45]. Due to high heterogeneity (I^2^: 92%, P_Q_ < 0.001), the random-effects model was implemented. Vasopressor use is associated with AKI in patients who receive vasopressors compared to those who do not receive vasopressor therapy [RR (95%CI): 3.17 (2.21, 4.54); *p* < 0.001] (Fig. 3).

**Fig. 4:**
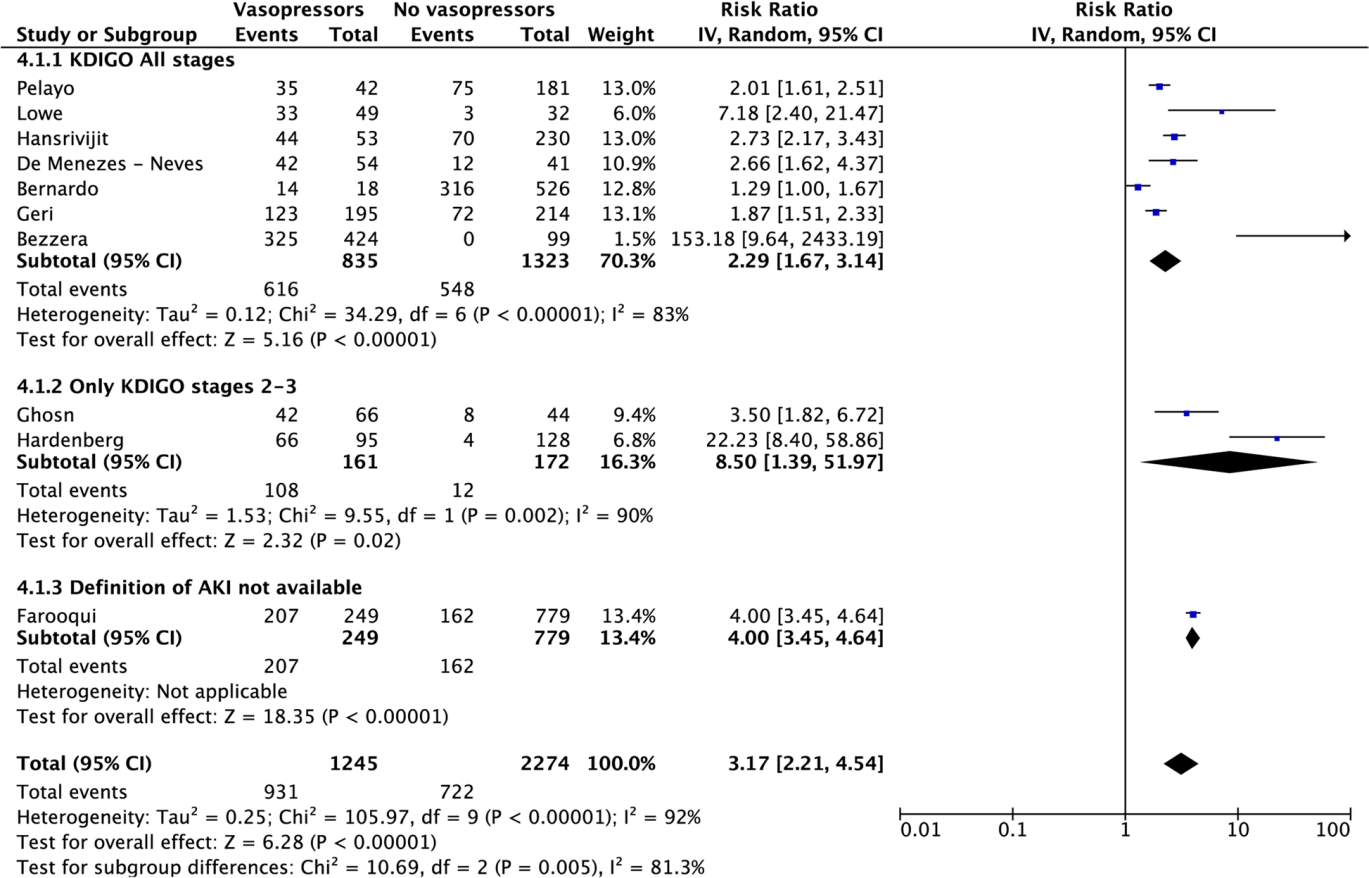
Subgroup analyses based on the definition of acute kidney injury

Subgroup analyses were performed based on the definition of AKI that was used in the included studies. Only the subgroup with patients at all KDINGO stages included more than three studies, allowing for meta-analysis to be performed. Specifically, vasopressor use is associated with AKI in patients who receive vasopressors compared to those who do not receive vasopressor therapy [RR (95%CI): 2.29 (1.67–3.14); *p* < 0.001] (Fig. 4).

#### Other secondary outcomes

No data were identified for the remaining secondary outcomes.

#### Sensitivity analysis

A sensitivity analysis was performed for both outcomes based on the implemented meta-analyses model (fixed *vs.* random effect); in both cases, the sensitivity analysis confirmed the robustness of the findings. The synthesized results of the present systematic review and meta-analysis together with the results of the sensitivity analysis are depicted in Table 3.

**Table 3:** Synthesis of the results with a sensitivity analysis (fixed *vs.* random effects)

Outcomes	RR (CI); *p* value Model used	Sensitivity analysis
		Alternate model
Mortality (all timepoints)	4.30 (3.21, 5.75); < 0.0001 Random effects	2.51 (2.39, 2.64); < 0.0001 Fixed effects
Mortality: subgroups based on department		
ICU	3.64 (2.44, 5.44); < 0.0001 Random effects	1.94 (1.78, 2.12); < 0.0001 Fixed effects
HDU	6.25 (4.63, 8.44); < 0.0001 Random effects	6.16 (4.81, 7.90); < 0.0001 Fixed effects
ICU & HDU	5.52 (2.51, 12.15); < 0.0001 Random effects	2.71 (2.55, 2.89); < 0.0001 Fixed effects
Mortality: subgroups based on mortality timepoints		
In hospital	4.59 (2.61, 8.10); < 0.0001 Random effects	2.30 (2.10, 2.52); < 0.0001 Fixed effects
In ICU	4.07 (2.28, 7.28); < 0.0001 Random effects	4.07 (2.28, 7.28); < 0.0001 Fixed effects
30-day	2.97 (1.72, 5.14); < 0.0001 Random effects	2.40 (2.25, 2.57); < 0.0001 Fixed effects
90-day	2.78 (1.98, 3.91); < 0.0001 Random effects	2.74 (2.27, 3.31); < 0.0001 Fixed effects
6-weeks	4.25 (2.26, 7.99); < 0.0001 Random effects	4.25 (2.26, 7.99); < 0.0001 Fixed effects
Not available	7.94 (5.72, 311.02); < 0.0001 Random effects	8.09 (6.16, 10.62); < 0.0001 Fixed effects
AKI (all timepoints)	3.17 (2.21, 4.54); < 0.0001 Random effects	2.64 (2.42, 2.88); < 0.0001 Fixed effects
AKI: subgroups based on KDIGO stage		
KDIGO all stages	2.29 (1.67, 3.14); < 0.0001 Random effects	2.02 (1.81, 2.26); < 0.0001 Fixed effects
KDIGO stages 2–3	8.50 (1.39, 51.97); 0.02 Random effects	6.21 (3.61, 10.68); < 0.0001 Fixed effects
Not available KDIGO	4.00 (3.45, 4.64); < 0.0001 Random effects	4.00 (3.45, 4.64); < 0.0001 Fixed effects

*ICU* Intensive Care Unit, *HDU* High Dependency Unit, *AKI* Acute Kidney Injury

### Risk of bias, quality of evidence

The overall quality of the studies, as assessed by the MINORS tool, ranged between moderate and high. The exact score for each study is available in Additional file 3: Appendix C3. In addition, visual inspection of the funnel plot (Additional file 4: Appendix D2) and the Begg’s test (*p* = 0.18) did not reveal significant publication bias for the studies included in the AKI analysis. All included studies were observational non-randomized studies with high heterogeneity that does not allow to derive an estimate of overall effect. According to GRADE criteria, the quality of evidence provided by the studies was low.

## Discussion

Many high-quality RCTs have addressed the effect of vasopressors on the outcomes of non-covid patients, yet their impact in patients with COVID-19 had not been studied so far. The most important finding of this systematic review and meta-analysis is the association between vasopressor therapy and in-hospital mortality, 30-day mortality, and incidence rate of AKI as compared to no vasopressor therapy in critically ill patients with COVID-19. Although these results are based on non-randomized evidence, they raise significant concerns for the routine management of these individuals.

The mortality of critically ill patients with COVID-19 remains high [55, 56]. A main cause is the characteristics of the SARS-CoV-2 infection, which can rapidly affect many organs including the cardiovascular system [57]. Although administration of vasopressors is a fundamental treatment of hypotension, the traditional (non-covid) hemodynamic management and the adverse effects of vasoactive agents may be associated with complications and poor outcome in patients with COVID-19. Indeed, the present analysis revealed an association between vasopressor use and mortality. This association may be coincidental due to the severity of critical illness. However, catecholamines exert numerous biological effects including effects on the immune and hematological systems, the renin–angiotensin–aldosterone system, the cardiovascular and respiratory systems, and others [9, 14]. This complex interplay that may have a detrimental effect on survival.

Of the 34 studies included in this systematic review and meta-analysis, only four studies provided data on specific vasopressors. The highest mortality rate was observed in patients receiving vasopressin or epinephrine. Although one can appraise that these patients had severe shock necessitating second- and third-line vasopressors [7], these observations merit further discussion. Epinephrine is well-known for its adverse effects in non-covid and COVID-19 patients [58], but our observations regarding vasopressin are quite interesting. Although vasopressin infusion reduces total norepinephrine-equivalent dose requirements and may be renal and pulmonary vasculature sparing [59], there is evidence showing a pronounced activation of the vasopressin system in COVID-19 patients and that molecular complexes form between the SARS-CoV-2 spike protein, soluble angiotensin-converting enzyme-2 (ACE2), and vasopressin, facilitating cellular infection and aggravating outcome [60, 61]. However, data from a small clinical cohort did not show a clinically relevant effect of vasopressin infusion on viral mRNA levels in critically ill patients with COVID-19 who were not treated with corticosteroids or interleukin-6 antagonists [59]. Considering the high heterogeneity of the extracted data in the present study and that vasopressin is suggested as a second-line vasopressor in the latest international guidelines [7, 9], further research is required to establish the therapeutic efficiency of vasopressin in critically ill patients with COVID-19.

Another intriguing finding is the low mortality rate in patients receiving angiotensin-II as a sole or second-line vasopressor agent. Serpa Neto et al. showed a potentially positive effect of angiotensin-II on blood pressure and fraction of inspired oxygen in COVID-19 patients, but they did not collect data regarding treatment with steroids or other drugs, which may have affected their results [50]. Ofosu-Barko et al. and Leisman et al. reported that angiotensin-II treatment was associated with rapid improvement in multiple physiologic indices [39, 52]. The rationale for angiotensin-II therapy is based on decreasing the expression of the ACE2 receptors, which facilitate the entry of SARS-CoV-2 into cells [62, 63]. Of note, the progressive loss of ACE2 in COVID-19 shifts the system to an overall higher angiotensin level due to the impaired ability of ACE2 to degrade it, which may explain the hemodynamic stability during the initial stages of the disease [64]. Moreover, recent experimental evidence suggests that angiotensin-II administration is associated with a similar level of cardiovascular resuscitation, less myocardial oxygen consumption, and less inflammation compared to norepinephrine [65]. Taking into consideration the characteristics of angiotensin-II, more research is needed to evaluate its potent effects in COVID-19-related shock.

A meta-analysis of RCTs with non-covid patients reported that vasopressor therapy is not associated with differences in mortality in the overall population, while prophylactic administration in patients with vasodilatory shock may improve survival [66]. In addition, a Cochrane systematic review found no evidence of substantial differences in total mortality between several vasopressors [67]. Nevertheless, vasopressors are a heterogeneous class of drugs with powerful and immediate hemodynamic effects, and each drug has advantages and disadvantages. These characteristics are particularly important in patients with COVID-19 who are characterized by unique pathophysiological disturbances and different hemodynamic phenotypes that necessitate a thorough understanding of the underlying complex pathophysiology and careful selection and administration of vasoactive agents.

In COVID-19, the progressive hypoxemia initially increases cardiac output and capillary recruitment, which maintain microcirculatory oxygen-extraction capacity by increasing red blood cell availability (silent hypoxia) [68–70]. However, microcirculatory flow decreases proportionally to the increasing inflammation, hypercoagulation, and thrombosis, eventually resulting in multi-organ failure [68, 71–73]. In the study by Mesquida et al., patients had important microcirculatory alterations, and the degree of these alterations correlated with the severity of the respiratory disease [25]. The relationship between MAP and organ blood flow may be different in critically ill patients with COVID-19 and improving only macrocirculation might be inadequate to maintain tissue perfusion. In these patients, vasopressor use can overwhelm endogenous receptor-mediated vessel regulation, further contributing to hemodynamic incoherence [71, 74], and therefore, hemodynamic management should focus on optimizing microcirculatory perfusion and oxygen delivery instead of attaining a predefined MAP target.

## Limitations

This meta-analysis was based on observational studies, while the results are subject to confounding by indication. In addition, due to the lack of RCTs, the synthesis of all the available knowledge on the specific outcomes was difficult. This is an inherent problem to observational studies, and not least considering the difficulties of collecting data during the periods of surges across the globe. Moreover, we could not obtain individual data to reach a minimal level of evidence that could result on relevant findings. Therefore, the effects of different vasopressors may reflect differences in severity and/or practices.

Furthermore, most of the included studies were published before November 2021 and thus, we were not able to analyze the data according to COVID-19 surge. In addition, the level of heterogeneity was high; possible reasons are the baseline status of patients, comorbidities, severity of COVID-19, and hospital department, i.e., HDU, ICU, and ED. There were no data for adjusting the resulting odds ratios according to age, comorbidities, the presence of septic shock, or other known factors that affect ICU mortality. Also, most of the secondary outcomes could not be assessed. Another limitation is the heterogeneity of definitions of AKI that were used across different studies. Finally, non-English publications were not included. Therefore, the results of this systematic review and meta-analysis must be interpreted with caution. International registries should collect uniform data to evaluate the effect of vasopressors on mortality and other outcomes in critically ill patients with COVID-19.

## Conclusions

Current use of vasopressors in critically ill patients with COVID-19 may be associated with higher in-hospital mortality, 30-day mortality, and incidence rate of AKI. The lower mortality rate in patients receiving angiotensin-II as a sole or second-line vasopressor agent is worth noting. Of note, the included studies were observational with high heterogeneity, which does not allow to derive an estimate of overall effect. Randomized controlled trials and translational research are required to estimate the correlation of specific vasopressor characteristics (type, timing, dose, combination) with adverse effects and mortality in this population.

## Perspectives

The results of the present systematic review and meta-analysis suggest for early administration of low-dose vasopressors, with or without inodilator agents, in an effort to avoid excessive doses that could have detrimental effect on survival, especially at later disease stages. An alternative second-line vasopressor may be angiotensin-II. However, further immediate research is recommended to elucidate the effects of angiotensin-II and other vasopressors acting through pathways other than the adrenergic. These agents may be associated with a significant increase in survival.

A possible explanation for the association of vasopressors with mortality may lie in the microcirculation [68–77]. The physiological pulsatile shear stress from normal laminar flow has a pivotal role in maintaining normal endothelial function and the expression of ACE2s and other anticoagulant/antithrombotic or antioxidant substances [78]. However, dysfunctional endothelium resulting from turbulent flow displays a hypercoagulant/prothrombotic and pro‐oxidant state that impairs microcirculatory reactivity and flow [79]. Therefore, therapeutic approaches should consider the systemic vascular involvement, allowing an individualized, physiology-guided management. It is almost certain that there are distinct COVID-19 phenotypes/subphenotypes that include impairment of microvasculature as key feature, and their identification will have important therapeutic implications [80]. Of note, a high CVP in critically ill patients with COVID-19 impairs venous return and retrogradely increases post-capillary venular pressure which, together with the excessive vasopressor doses, impair capillary perfusion and increase the oxygen diffusion distance [81, 82]. Consequently, optimizing fluid administration is also crucial for improving tissue perfusion in this population.

The present systematic review and meta-analysis included data from observational studies. Further research and well-designed trials are necessary to investigate the effect of the type (catecholamine *vs.* non-catecholamine), timingof initiation, and infusion rates of vasopressors in order to develop more specific treatment strategies and integrate a more individualized approach in patients with COVID-19. Although designing and conducting RCTs on vasopressors may be difficult during a disease outbreak, the need for assessing their effect on outcomes of critically ill patients with COVID-19 is imperative. We recommend the use of animal models and the integration of translational research to aid in the identification of the most suitable vasopressor in this population and to better define homogenous target (sub)populations for trials [83–85]. Large pragmatic RCTs with very broad inclusion criteria can help improving the generalizability of our findings.

## Supplementary Information

**Additional file 1:** **Appendix A.** PRISMA checklist.

**Additional file 2: Appendix B.** Algorithms used for all databases.

**Additional file 3: Appendix C1.** Definitions used for AKI and mortality follow-up time points. **Appendix C2.** Angiotensin-related data. **Appendix C3.** MINORS results for each study.

**Additional file 4: Appendix D1.** Funnel plot for mortality meta-analysis. **Appendix D2.** Funnel plot for AKI meta-analysis.

## Data Availability

Data can be made available upon request after publication through a collaborative process. Researchers should provide a methodically sound proposal with specific objectives in an approval proposal. Please contact the corresponding author for additional information.

## Abbreviations

COVID-19: Coronavirus disease 2019
RR: Risk ratios
CI: Confidence interval
ARDS: Acute respiratory distress syndrome
MAP: Mean arterial pressure
RCT: Randomized controlled trial
ICU: Intensive care unit
HDU: High dependency unit
ED: Emergency department
CVP: Central venous pressure
AKI: Acute kidney injury
MINORS: Methodological index fornon-randomized studies
ACE2: Angiotensin-converting enzyme 2
