# Supplementary material for: Association of vasopressors with mortality in critically ill patients with COVID-19: a systematic review and meta-analysis
Source: Anesthesiol Perioper Sci. 2023 Apr 23;1(2):10. doi: 10.1007/s44254-023-00013-7 (PMC10122723; doi:10.1007/s44254-023-00013-7)
Supplement: Supplementary file 2 — Additional file 2: Appendix B. Algorithms used for all databases. [file 44254_2023_13_MOESM2_ESM.docx]

PubMed: 862 results

**("vasopressor*" OR epinephrine[MeSH Terms] OR norepinephrine[MeSH Terms] OR phenylephrine[MeSH Terms] OR vasopressins[MeSH Terms] OR Dopamine[MeSH Terms] OR angiotensins[MeSH Terms]) AND (covid-19)** Filters: **English**

Scopus: 610 results

TITLE-ABS ( "vasopressor*"  OR  "epinephrine"  OR  "norepinephrine"  OR  "phenylephrine"  OR  "vasopressin*"  OR  "dopamine"  OR  "angiotensin" )  AND  TITLE-ABS-KEY ( "COVID-19" )  AND  TITLE-ABS-KEY ( "critically ill"  OR  icu  OR  "intensive care" )  AND  ( EXCLUDE ( SRCTYPE ,  "k" )  OR  EXCLUDE ( SRCTYPE ,  "p" ) )  AND  ( EXCLUDE ( DOCTYPE ,  "re" )  OR  EXCLUDE ( DOCTYPE ,  "le" )  OR  EXCLUDE ( DOCTYPE ,  "ed" )  OR  EXCLUDE ( DOCTYPE ,  "no" )  OR  EXCLUDE ( DOCTYPE ,  "sh" )  OR  EXCLUDE ( DOCTYPE ,  "cp" )  OR  EXCLUDE ( DOCTYPE ,  "ch" )  OR  EXCLUDE ( DOCTYPE ,  "tb" ) )  AND  ( EXCLUDE ( LANGUAGE ,  "French" )  OR  EXCLUDE ( LANGUAGE ,  "Russian" )  OR  EXCLUDE ( LANGUAGE ,  "Spanish" )  OR  EXCLUDE ( LANGUAGE ,  "Chinese" )  OR  EXCLUDE ( LANGUAGE ,  "Persian" )  OR  EXCLUDE ( LANGUAGE ,  "Portuguese" )  OR  EXCLUDE ( LANGUAGE ,  "German" )  OR  EXCLUDE ( LANGUAGE ,  "Hungarian" ) )

Clinicaltrials.gov: 22 results

**Completed Studies |** **(vasopressor OR epinephrine OR norepinephrine OR phenylephrine OR vasopressin OR Dopamine OR angiotensin) AND (covid-19)**
