# Supplementary figures and images for: Association of vasopressors with mortality in critically ill patients with COVID-19: a systematic review and meta-analysis
Source: Anesthesiol Perioper Sci. 2023 Apr 23;1(2):10. doi: 10.1007/s44254-023-00013-7 (PMC10122723; doi:10.1007/s44254-023-00013-7)

Appendix D1


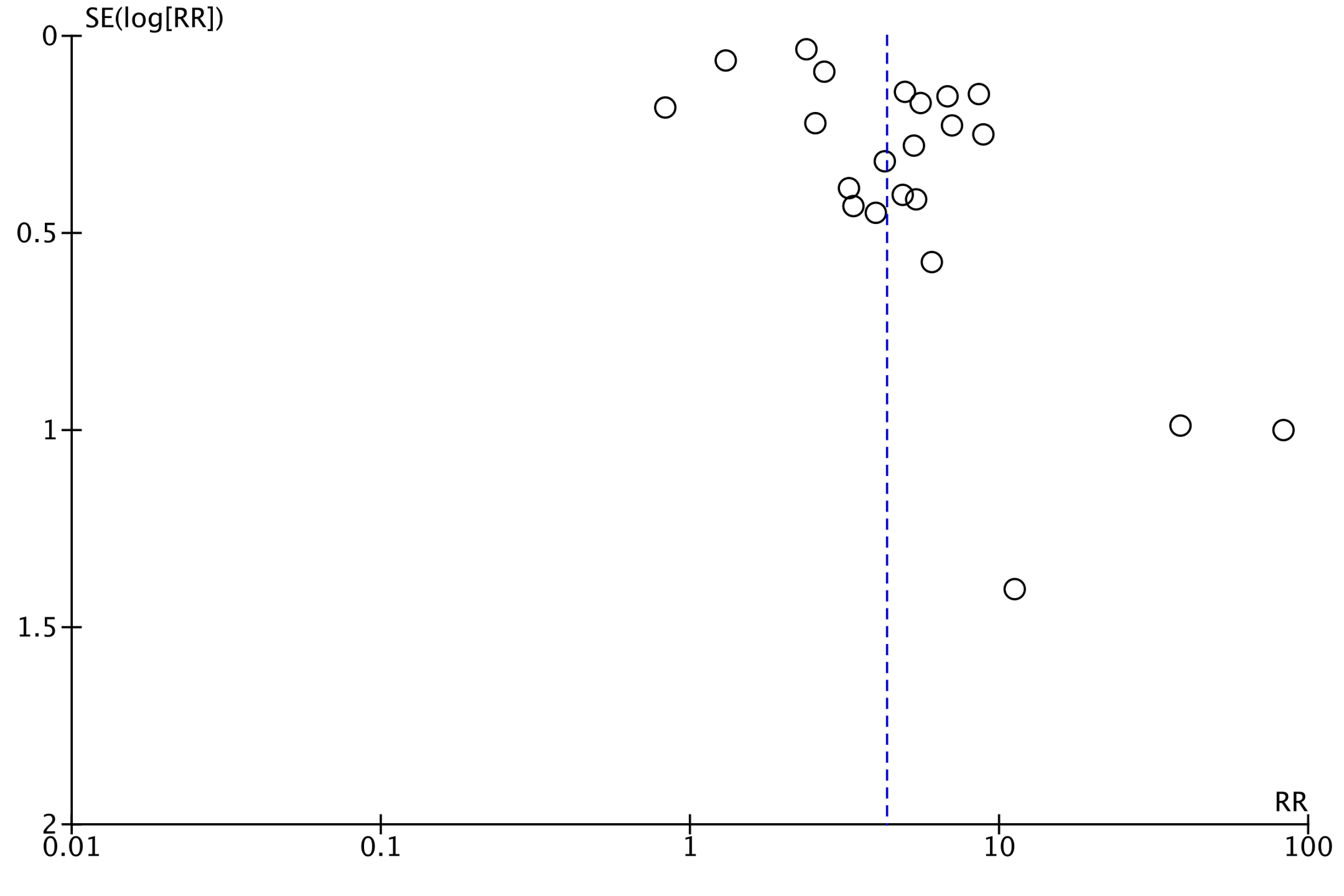


Appendix D2

Supplement: Supplementary file 4 — Additional file 4: Appendix D1. Funnel plot for mortality meta-analysis. Appendix D2. Funnel plot for AKI meta-analysis. [file 44254_2023_13_MOESM4_ESM.docx]
